# Edentulism, blood‐based neurodegenerative biomarkers, and 10‐year dementia risk: A mediation analysis in a nationally representative United States cohort

**DOI:** 10.1002/alz.71731

**Published:** 2026-08-05

**Authors:** Xiang Qi, Ruotong Liu, Daniel W. Belsky, Qu Tian, Huabin Luo, Yunrui Liu, Zhijing Xu, Kenneth M. Langa, Bei Wu

**Affiliations:** ^1^ Rory Meyers College of Nursing New York University New York New York USA; ^2^ Department of Epidemiology and Robert N Butler Columbia Aging Center Mailman School of Public Health Columbia University New York New York USA; ^3^ Longitudinal Studies Section (LSS), Translational Gerontology Branch, Intramural Research Program, National Institute on Aging National Institutes of Health Baltimore Maryland USA; ^4^ Brody School of Medicine, Department of Public Health East Carolina University Greenville North Carolina USA; ^5^ Department of Internal Medicine University of Michigan Ann Arbor Michigan USA; ^6^ NYU Shanghai Shanghai China

**Keywords:** Alzheimer's disease, neurodegenerationoral health, periodontitis, tooth loss

## Abstract

**INTRODUCTION:**

Tooth loss is associated with dementia risk, but mechanisms remain unclear. We examined whether blood‐based neurodegenerative biomarkers mediate the edentulism–cognition association.

**METHODS:**

Among 4238 Health and Retirement Study participants aged 50+, we linked edentulism (2012), biomarkers (neurofilament light chain [NfL], glial fibrillary acidic protein [GFAP], phosphorylated tau‐181, and amyloid‐β [Aβ] 42/40; 2016), and 10‐year cognitive outcomes (2012–2022).

**RESULTS:**

Edentulism was associated with higher NfL (*β =* 4.21) and GFAP (*β =* 8.06), but not pTau‐181 or Aβ42/40, and predicted cognitive impairment (hazard ratio [HR] 1.49) and dementia (HR 1.28). GFAP mediated 9.8% and 12.0% of the cognitive impairment and dementia associations, respectively; NfL mediated 9.8% of the dementia association but not cognitive impairment.

**CONCLUSION:**

In this observational cohort, elevated NfL and GFAP were associated with, and accounted for a small proportion of, the edentulism–cognition association. These associative findings support further study of oral health in dementia prevention but do not establish a causal effect.

## Highlights

- Edentulism was linked to higher plasma glial fibrillary acidic protein (NfL) and glial fibrillary acidic protein (GFAP), but not Alzheimer‐specific biomarkers.
- Edentulism predicted 10‐year incident cognitive impairment and dementia in older US adults.
- GFAP statistically mediated a small share of the edentulism–cognition associations.
- NfL mediated part of the edentulism–dementia association but not cognitive impairment.
- Findings are associative and point to a generalized neuroinflammatory, not amyloid, signature.

## 1 BACKGROUND

Dementia is among the most pressing public health challenges of the 21st century. An estimated 57 million people worldwide were living with dementia in 2021, and this number is projected to reach 153 million by 2050 as populations age.^1^ In the absence of disease‐modifying therapies for most forms of dementia, identifying and mitigating modifiable risk factors across the life course has become a central strategy for reducing the population burden of cognitive decline.^2^

The 2024 *Lancet* Commission on dementia prevention, intervention, and care identified 14 modifiable risk factors that collectively account for 45% of cases.^2^ Although oral health was not formally included as a standalone risk factor in the Commission's framework, a growing body of epidemiological evidence implicates poor oral health, and tooth loss in particular, as a potentially important contributor to cognitive decline.^3^, ^4^, ^5^ A recent correspondence in *The Lancet* argued that the population‐attributable fraction for edentulism (4.64%) exceeds that of several risk factors currently included in the framework.^6^ Meta‐analyses have consistently demonstrated that tooth loss is associated with a 28% to 48% increased risk of cognitive impairment and a 14% to 55% increased risk of dementia,^4^, ^7^, ^8^, ^9^ with evidence of a dose‐response relationship in which each additional missing tooth incrementally elevates risk.^4^, ^7^

Several biological mechanisms have been proposed but evidence has been limited by small sample sizes, convenience sampling, or reliance on animal models.^10^, ^11^, ^12^, ^13^ Chronic periodontal infections generate sustained systemic inflammation, with elevated circulating levels of C‐reactive protein, interleukin‐6, and tumor necrosis factor‐α, which can compromise the blood‐brain barrier and activate neuroinflammatory cascades.^12^, ^14^, ^15^, ^16^ Periodontal pathogens, notably *Porphyromonas gingivalis*, have been identified in postmortem Alzheimer's disease (AD) brains and shown to promote amyloid‐β (Aβ) production and neuroinflammation.^11^, ^17^, ^18^ Tooth loss also impairs masticatory function, leading to reduced intake of protective nutrients,^19^ and diminishes sensory input through the trigeminal nerve to the hippocampus, a structure critical for memory formation.^10^, ^20^ In addition, two recent UK Biobank studies have linked poor oral health to white matter injury^21^, ^22^ and shown that circulating proteomic markers partially mediate this association,^21^ although both examined neuroimaging markers of brain integrity rather than incident dementia.

Advances in ultrasensitive immunoassay technology have enabled reliable measurement of brain‐derived proteins in peripheral blood. Neurofilament light chain (NfL) is a cytoskeletal protein released upon neuroaxonal injury and validated as a sensitive marker of neurodegeneration.^23^ Glial fibrillary acidic protein (GFAP) reflects astrocyte reactivity; elevated plasma GFAP has been associated with amyloid pathology and cognitive decline in preclinical AD.^24^, ^25^ Phosphorylated tau‐181 (pTau‐181) and the Aβ 42/40 ratio reflect tau phosphorylation and amyloid metabolism, respectively, and are more specific to AD pathology.^26^ These biomarkers are increasingly used to detect preclinical neurodegeneration and to elucidate pathways linking modifiable risk factors to cognitive outcomes.^27^

**RESEARCH IN CONTEXT**

**Systematic review**: We searched PubMed and Embase from database inception to Jan 31, 2026, using the terms “edentulism” OR “tooth loss” AND “dementia” OR “cognitive impairment” AND “biomarkers” OR “neurofilament” OR “GFAP”, with no language restrictions. Meta‐analyses have shown that tooth loss is associated with a 28% to 48% increased risk of cognitive impairment and a 14% to 55% increased risk of dementia, with evidence of a dose‐response relationship. Several biological mechanisms have been proposed, including systemic inflammation from periodontal disease, direct microbial invasion of the brain, biomarker‐based biological aging, impaired masticatory function, and reduced trigeminal nerve stimulation of the hippocampus. However, no population‐based study had examined whether neurodegenerative biomarkers assessed in blood lie on the pathway between tooth loss, in particular edentulism, and incident cognitive impairment and dementia.

**Interpretation**: In this nationally representative cohort of older US adults, edentulism was independently associated with elevated plasma markers of neuroaxonal injury (neurofilament light chain) and astrocyte reactivity (glial fibrillary acidic protein), but not with Alzheimer‐specific biomarkers (phosphorylated tau‐181 and the amyloid‐β 42/40 ratio), suggesting a generalized neuroinflammatory rather than amyloid‐driven signature. Edentulism predicted 10‐year incident cognitive impairment and dementia, although the dementia association was attenuated and no longer statistically significant after full adjustment, particularly for dental care utilization. In counterfactual mediation analyses, glial fibrillary acidic protein (GFAP) and neurofilament light chain (NfL) each accounted for only a small proportion of these associations. Because the study is observational, these findings are associative and do not establish a causal or preventive effect.

**Future directions**: These results identify a candidate neuroinflammatory signature linking oral health to cognitive aging and motivate further study with serial biomarker measurements, clinical dental examinations, and designs better suited to causal inference. Because the edentulism–dementia association was largely explained by upstream socioeconomic and access‐related factors, future work should clarify whether oral health itself, or the social conditions it reflects, drives risk, and should pursue equitable access to dental care among the populations who bear the greatest burden of both edentulism and dementia.

Despite the epidemiological evidence and mechanistic plausibility, no study has examined whether blood‐based neurodegenerative biomarkers mediate the tooth loss, especially the edentulism–cognitive impairment association. Using data from the Health and Retirement Study (HRS), we conducted a population‐based mediation analysis to examine (1) the associations of edentulism with four blood‐based neurodegenerative biomarkers, (2) the association of edentulism with 10‐year risk of incident overall cognitive impairment or dementia, and (3) the extent to which of these neurodegenerative biomarkers mediate the edentulism–cognition association.

## 2 METHODS

### 2.1 Study design and population

This study used data from the HRS, a nationally representative longitudinal panel study of Americans aged 51 years and older, conducted biennially by the University of Michigan and sponsored by the National Institute on Aging. The HRS includes oversamples of Black and Hispanic populations and collects extensive survey data on health, cognition, and socioeconomic factors.^28^ The HRS protocol was approved by the Institutional Review Board at the University of Michigan (approval number HUM00099822); all participants provided written informed consent.

**FIGURE 1:**
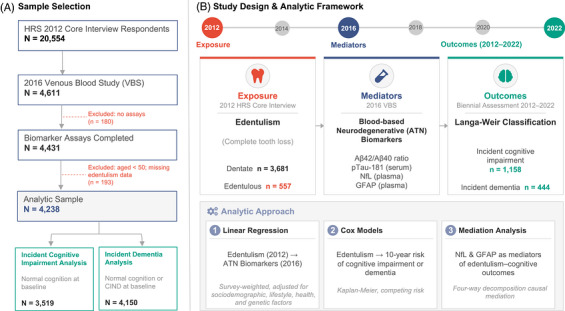
Sample selection and overview of the study design, Health and Retirement Study (HRS) 2012‐2022

This analysis linked data from three HRS components: (1) the 2012 core interview, which provided baseline oral health status and cognitive assessments; (2) the 2016 Venous Blood Study (VBS), which provided blood‐based biomarker measurements; and (3) biennial cognitive assessments from 2012 through 2022 (Figure 1A depicts study design). The 2016 VBS involved a probability subsample of HRS respondents born in 1959 or earlier who completed both a core interview and a venous blood draw in 2016. Neuropathological biomarkers were assayed on 4,431 participants within this subsample.^29^ Participants were included if they had valid data on edentulism in 2012, at least one biomarker measurement from the 2016 VBS, and cognitive assessment data from 2012 onward.^29^ Participants with missing data on edentulism or all biomarker measurements were excluded. The final analytic sample comprised 4,238 participants (sample selection flowchart in Figure 1B). Compared with the 16,316 excluded participants, included participants were modestly younger, more likely to be married and college educated, and somewhat healthier across several indicators (see Table S1 for detailed comparison).

### 2.2 Measures

#### 2.2.1 Edentulism

Edentulism was ascertained from the 2012 HRS core interview based on self‐reported complete tooth loss. Participants were classified as either dentate (retaining at least one natural tooth) or edentulous (having lost all natural teeth). The 2012 wave was selected as the baseline because it preceded the 2016 VBS, ensuring that the exposure was measured before the biomarker mediators, and because the edentulism item was administered in that wave.

#### 2.2.2 Blood‐based biomarkers of neurodegeneration

Four blood‐based biomarkers of neurodegeneration were measured as part of the 2016 HRS VBS. Blood was collected from consenting participants in their homes by certified phlebotomists and shipped overnight in temperature‐controlled packages to the Advanced Research and Diagnostic Laboratory at the University of Minnesota, with 92% of samples arriving within 24 hours and 99% within 48 hours of collection.^30^ Aβ42, Aβ40, NfL, and GFAP were measured in EDTA plasma using the Quanterix Neurology 4‐Plex E kit on an HD‐X analyser, and pTau‐181 was measured in serum using the Quanterix pTau‐181 Advantage V2.0 kit on the same platform.^31^ The Aβ42/40 ratio was calculated as a marker of amyloid pathology. All assays were performed in three batches using the same reagent lot, and the interassay coefficients of variation ranged from 8.55% to 13.34% based on pooled controls.^29^

#### 2.2.3 Cognitive impairment and dementia

Cognitive status was classified at each biennial HRS wave (2012 to 2022) using the Langa‐Weir Classification of Cognitive Function,^32^ a validated algorithmic approach widely used in population‐based research.^32^, ^33^ For self‐respondents, cognitive function was assessed using items from the modified Telephone Interview for Cognitive Status (TICS‐m), including immediate and delayed 10‐word recall (0 to 20 points), serial sevens subtraction (0 to 5 points), and backward counting from 20 (0 to 2 points), yielding a total score ranging from 0 to 27. Respondents were classified as having normal cognition (12 to 27 points), cognitive impairment no dementia (CIND; 7 to 11 points), or dementia (0 to 6 points). For proxy respondents, an alternative scoring system was used. Incident overall cognitive impairment was defined as a transition from normal cognition at baseline (2012) to CIND or dementia at any subsequent wave through 2022 (10‐year follow‐up). Incident dementia was defined as a transition from normal cognition or CIND at baseline to dementia at any subsequent wave through 2022.

#### 2.2.4 Covariates

Covariates were selected a priori based on existing literature.^3^, ^4^, ^5^, ^12^, ^34^ Sociodemographic variables included age, sex, race and ethnicity, marital status, education, and annual household income. Behavioral factors included smoking status, physical exercise, obesity, and dental care utilization, measured as a self‐reported dental visit within the past 2 years. Chronic conditions included depression, hypertension, diabetes, heart disease, arthritis, and stroke, all based on self‐report of a physician diagnosis. Apolipoprotein E (APOE) ε4 carrier status was determined from genotyping of salivary DNA or imputed from genotyped array data in HRS. The directed acyclic graph is shown in Figure S1.

### 2.3 Statistical analysis

All analyses incorporated survey weights from the 2016 VBS to account for the complex sampling design and differential nonresponse. The association of edentulism with each blood‐based biomarker was examined using survey‐weighted linear regression models with progressive covariate adjustment. Model 1 was unadjusted. Model 2 adjusted for sociodemographic factors. Model 3 additionally adjusted for health behaviors and clinical conditions. Model 4 further adjusted for APOE ε4 status.

Cox proportional hazards models estimated 10‐year hazard ratios (HRs) for cognitive outcomes with the same sequential covariate adjustment. Time to event was measured from the 2012 interview date to the midpoint between the last unimpaired assessment and the first assessment meeting outcome criteria. Participants without the outcome were censored at their last available cognitive assessment or date of death, whichever came first. Cause‐specific Cox proportional hazards models and Fine–Gray competing risk regression models accounted for the competing risk of death.

The mediation analysis used the counterfactual four‐way decomposition for survival outcomes. STATA *med4way* package was used to estimate natural direct and natural indirect effects on the hazard‐ratio scale with 1,000 bootstrap confidence intervals and jointly modeling the edentulism by mediator interaction.^35^ The total effect was partitioned into the controlled direct effect (CDE), the reference interaction (INTref), the mediated interaction (INTmed), and the pure indirect effect (PIE). Identification required four assumptions: no unmeasured exposure‐outcome, mediator‐outcome, or exposure‐mediator confounding, and no mediator‐outcome confounder affected by the exposure.^36^, ^37^, ^38^, ^39^ Path a estimated the edentulism‐mediator association using weighted linear regression. Path b estimated the mediator‐outcome association using a Cox model adjusting for edentulism. The proportion mediated was calculated as ((total β − direct β) / total β) × 100, where β values represent the log‐hazard ratio coefficients. We focused on NfL and GFAP as candidate mediators based on results from the biomarker analyses, which demonstrated significant associations of edentulism with these two markers. Mediation models were conducted using the same sequential covariate adjustment.

Several sensitivity analyses were performed. First, interaction terms between edentulism and APOE ε4 and between edentulism and baseline cognitive status were examined. Second, to address the temporal ordering of exposure, mediator, and outcome, a landmark analysis reset the time origin to 2016, retained only participants event‐free through 2016, and restricted follow‐up to 2016 through 2022. Third, to assess robustness to unmeasured confounding, E‐values were computed for the exposure–outcome and mediator–outcome associations.^40^ Fourth, to probe the influence of dental care utilization, models were stratified by dental care status and the edentulism–cognition associations by dental care interaction were tested. Fifth, because the Langa‐Weir algorithm is less accurate in minoritized populations, the edentulism–dementia association was re‐estimated using the race‐and‐ethnicity‐aware Gianattasio‐Power dementia classifications.^41^ Sixth, a multiple imputation analysis (20 imputations by chained equations) addressed item‐level covariate missingness. Proportional‐hazards assumptions were assessed using Schoenfeld residuals. All statistical tests were two‐sided with a significance level of *p <* 0.05. Reporting followed the AGReMA^42^ and STROBE guidelines, and the completed checklists are provided as supplementary files. Analyses were conducted using Stata/MP version 19.5 (StataCorp, 2025) and R (Version 4.5.2; R Core Team, 2025).

## 3 RESULTS

### 3.1 Sample characteristics

The analytic sample included 4,238 participants, of whom 557 were edentulous and 3,681 were dentate (Table 1). Compared with dentate participants, edentulous participants were older, more likely to be non‐Hispanic Black individuals, unmarried, and had lower educational attainment and household income. Edentulous participants had higher rates of current smoking, were less likely to exercise, and had higher prevalence of depression, hypertension, diabetes, heart disease, arthritis, and stroke. Only 25.5% of edentulous participants reported a dental visit in the past 2 years compared with 70.6% of dentate participants. The prevalence of APOE ε4 carriers did not differ significantly between groups.

**TABLE 1:** Baseline characteristics by edentulism status, Health and Retirement Study 2012 (*N =* 4,238)

Characteristic	Edentulous (*n =* 557)	Dentate (*n =* 3,681)	Total (*N =* 4,238)	SMD	*p*‐Value
Age, mean (SD)	68.6 (9.7)	64.7 (9.5)	65.2 (9.6)	0.41	<0.001
Age groups, n (%)					<0.001
≤64	210 (37.7)	2,018 (54.8)	2,228 (52.6)	−0.35	
65–74	180 (32.3)	989 (26.9)	1,169 (27.6)	0.12	
75+	167 (30.0)	674 (18.3)	841 (19.8)	0.28	
Female, n (%)	323 (58.0)	2,167 (58.9)	2,490 (58.8)	−0.02	0.728
Race and ethnicity, n (%)					0.021
Non‐Hispanic White	346 (62.1)	2,449 (66.5)	2,795 (66.0)	−0.09	
Non‐Hispanic Black	119 (21.4)	593 (16.1)	712 (16.8)	0.13	
Hispanic	74 (13.3)	524 (14.2)	598 (14.1)	−0.03	
Other	18 (3.2)	115 (3.1)	133 (3.1)	0.01	
Married, n (%)	316 (56.7)	2,552 (69.3)	2,868 (67.7)	−0.26	<0.001
Education, n (%)					<0.001
Low (≤high school)	422 (75.8)	1,691 (45.9)	2,113 (49.9)	0.64	
Mid (some college)	98 (17.6)	1,000 (27.2)	1,098 (25.9)	−0.23	
High (college+)	37 (6.6)	990 (26.9)	1,027 (24.2)	−0.56	
Annual household income, n (%)					<0.001
< $50,000	435 (78.1)	1,887 (51.3)	2,322 (54.8)	0.58	
$50,000–$99,999	95 (17.1)	1,006 (27.3)	1,101 (26.0)	−0.25	
$100,000–$199,999	20 (3.6)	560 (15.2)	580 (13.7)	−0.41	
≥$200,000	7 (1.3)	228 (6.2)	235 (5.5)	−0.26	
Dental visit past 2 years, n (%)	142 (25.5)	2,598 (70.6)	2,740 (64.7)	−1.01	<0.001
Smoking status, n (%)					<0.001
Never	146 (26.2)	1,727 (46.9)	1,873 (44.2)	−0.44	
Former	268 (48.1)	1,507 (40.9)	1,775 (41.9)	0.14	
Current	143 (25.7)	447 (12.1)	590 (13.9)	0.35	
Exercise (≥1/wk), n (%)	342 (61.4)	2,691 (73.1)	3,033 (71.6)	−0.25	<0.001
Obesity (BMI≥30), n (%)	212 (38.1)	1,444 (39.2)	1,656 (39.1)	−0.02	0.631
Depression, n (%)	128 (23.0)	519 (14.1)	647 (15.3)	0.23	<0.001
Hypertension, n (%)	363 (65.2)	2,074 (56.3)	2,437 (57.5)	0.18	<0.001
Diabetes, n (%)	174 (31.2)	761 (20.7)	935 (22.1)	0.24	<0.001
Heart disease, n (%)	153 (27.5)	696 (18.9)	849 (20.0)	0.20	<0.001
Arthritis, n (%)	375 (67.3)	2,001 (54.4)	2,736 (56.1)	0.22	<0.001
Stroke, n (%)	73 (13.1)	219 (5.9)	292 (6.9)	0.25	<0.001
APOE ε4 carrier, n (%) [*n =* 3,829]	135 (26.9)	846 (25.4)	981 (25.6)	0.03	0.500
Follow‐up for cognitive impairment, years, mean (SD)	7.0 (3.0)	8.1 (2.7)	8.0 (2.8)	0.38	<0.001
Follow‐up for dementia, years, mean (SD)	8.3 (2.2)	9.0 (1.9)	8.9 (2.0)	0.34	<0.001

*Note*: Counts n (%) are presented for categorical variables and mean (SD) for continuous variables. *p*‐Values are from chi‐squared tests (categorical) or independent t‐tests (continuous). Standardized mean differences (SMD) between the dentate and edentulous groups are also reported. The APOE ε4 percentages are computed among genotyped participants only (*n =* 3,829).

Abbreviations: APOE, apolipoprotein E; BMI, body mass index.

### 3.2 Associations of edentulism with blood‐based neurodegenerative biomarkers

**FIGURE 2:**
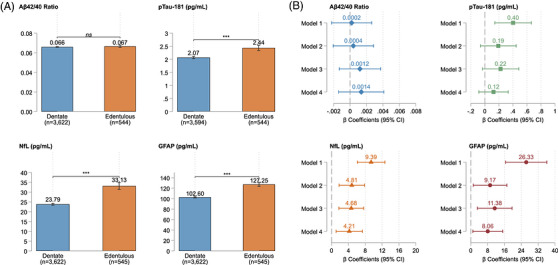
(a) Unweighted mean blood‐based neurodegenerative biomarkers by edentulism status (2012). Bars represent unweighted means; error bars show standard error of the mean (SEM). ***p < 0.001; ns, not significant. (b) Forest plot of the association of edentulism with blood‐based neurodegenerative biomarkers. Model 1: unadjusted; Model 2: adjusted for age, gender, race/ethnicity, marital status, education, income, and dental care utilization; Model 3: Model 2 + smoking, exercise, obesity, depression, hypertension, diabetes, heart disease, and stroke; Model 4: Model 3 + apolipoprotein E (APOE) ε4. All models weighted by the Venous Blood Study survey weight. NfL, neurofilament light chain; GFA*O*, glial fibrillary acidic protein; pTau‐181 = phosphorylated tau‐181

Edentulism was significantly associated with higher NfL and GFAP levels across all models (Figure 2; Table S2). In Model 1 that did not adjust for any covariates, edentulous participants had, on average, 9.39 pg/mL higher NfL levels (95% confidence interval [CI] 6.12 to 12.67, *p <* 0.001) and 26.33 pg/mL higher GFAP levels (16.55 to 36.11, *p <* 0.001) than dentate participants. After full adjustment (Model 4), edentulism remained significantly associated with higher NfL (*β =* 4.21, 95% CI 1.08 to 7.33, *p =* 0.008) and higher GFAP (*β =* 8.06, 0.97 to 15.15, *p =* 0.026). Edentulism was associated with higher serum pTau‐181 in the unadjusted Model 1 (*β =* 0.40, 0.14 to 0.66, *p =* 0.002) but this was no longer significant after sociodemographic adjustment (Model 2: *β =* 0.19, ‐0.06 to 0.45, *p =* 0.140). No association was observed with Aβ 42/40 in any model (all *p >* 0.05).

### 3.3 Association of edentulism with incident overall cognitive impairment and dementia

**FIGURE 3:**
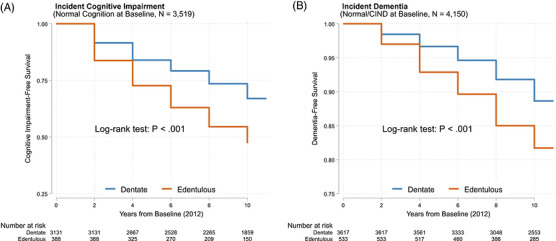
Kaplan–Meier survival curves by edentulism status (Health and Retirement Study 2012–2022)

Among 3,519 participants with normal cognition at baseline, 1,163 developed incident overall cognitive impairment over 10 years (Figure 3A). In the unadjusted model, edentulism was associated with more than double the hazard of incident cognitive impairment (HR 2.03, 95% CI 1.77 to 2.33, *p <* 0.001; Table S3). This was progressively attenuated with covariate adjustment but remained significant in Model 4 (HR 1.49, 1.20 to 1.85, *p <* 0.001). Among 4,150 participants without dementia at baseline, 455 developed incident dementia (Figure 3B). Edentulism was associated with a twofold increase in the unadjusted model (HR 2.04, 1.62 to 2.57, *p <* 0.001). After full adjustment (Model 4), the association remained significant (HR 1.28, 1.03 to 1.59, *p =* 0.025; Table S3).

Edentulism was also associated with higher 10‐year all‐cause mortality (Model 4: HR 1.40, 1.12 to 1.75, *p <* 0.001; Table S4). In cause‐specific Cox models accounting for the competing risk of death, edentulism remained significantly associated with both incident cognitive impairment (cause‐specific HR 1.22, 1.02 to 1.46, *p =* 0.030) and incident dementia (cause‐specific HR 1.28, 1.08 to 1.51, *p =* 0.004) in the fully adjusted models (Table S5). Fine‐Gray competing risk regression yielded similar results (cognitive impairment: HR 1.19, 1.06 to 1.34, *p =* 0.004; dementia: HR 1.21, 1.03 to 1.45, *p =* 0.021).

### 3.4 Mediation analysis

**FIGURE 4:**
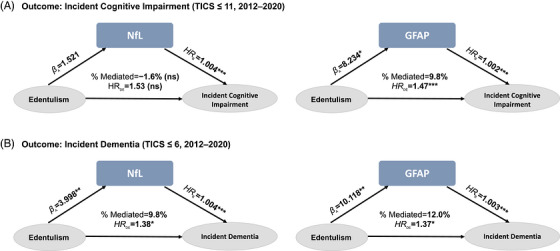
Four‐way decomposition causal mediation analysis: neurofilament light chain (NfL) and glial fibrillary acidic protein (GFAP) as mediators of edentulism–cognitive outcome associations. The displayed natural direct and natural indirect effects from the four‐way decomposition. βA, path a (edentulism → mediator, linear regression); HRB, path b (mediator → outcome, Cox model); HRDE, direct effect. All models adjusted for age, sex, race and ethnicity, marital status, education, income, dental care utilization, smoking, exercise, obesity, depression, hypertension, diabetes, heart disease, arthritis, stroke, and apolipoprotein E (APOE) ε4. *p < 0.05; **p < 0.01; ***p < 0.001; ns = not significant. A negative proportion mediated arises when the direct‐effect hazard ratio slightly exceeds the total‐effect hazard ratio, which can occur when the indirect path is near zero and is consistent with negligible mediation rather than a suppression effect

Four‐way decomposition causal mediation analyses assessed the extent to which NfL and GFAP explained the edentulism–cognitive impairment and edentulism–dementia associations (Figure 4). The decomposition reproduced the overall pattern of the primary mediation findings, with a small mediated proportion; the edentulism by mediator interaction was not statistically significant for most pathways, although possible nominal interaction was observed for the GFAP and cognitive impairment pathway and the NfL and dementia pathway, which are reported in full in Table S6.

In the fully adjusted Model 4, the total effect of edentulism on incident cognitive impairment was HR 1.52 (95% CI 1.24 to 1.86). GFAP partially mediated this association, accounting for 9.8% of the total effect, with a significant path a (edentulism to GFAP: *β =* 8.234, *p =* 0.031) and path b (GFAP to cognitive impairment: HR 1.0022, *p <* 0.001). NfL did not significantly mediate the cognitive impairment association (proportion mediated ‐1.6%; path a *β =* 1.521, *p =* 0.269).

For incident dementia, the total effect was HR 1.42 (95% CI 1.04 to 1.94), and both NfL and GFAP were significant mediators. NfL mediated 9.8% of the association, with significant path a (*β =* 3.998, *p =* 0.005) and path b (HR 1.0039, *p <* 0.001) coefficients, and GFAP mediated 12.0%, with significant path a (*β =* 10.118, *p =* 0.016) and path b (HR 1.0025, *p <* 0.001) coefficients.

Per‐analyte distributions, interassay coefficients of variation, and post‐hoc power are summarized in Table S7. All four biomarkers were right‐skewed (raw skewness 2.4 to 11.6) and were natural‐log transformed for analysis, which reduced skewness to within ± 0.6; per‐analyte interassay coefficients of variation ranged from 8.6% to 13.3%, and the analysis had 80% power to detect a standardized mean difference of 0.13 for pTau‐181 and Aβ42/40.

### 3.5 Sensitivity analyses

First, interaction analyses revealed no significant effect modification by APOE ε4 status on any biomarker association (Table S8). Similarly, CIND or dementia status did not modify the edentulism‐biomarker associations (Tables S9 to S10). Second, of the incident events, 608 cognitive impairment events and 159 dementia events occurred at or before the 2016 biomarker wave. In the landmark analysis resetting the time origin to 2016 (Table S11), the direction of the unadjusted associations was preserved, although after full adjustment the cognitive impairment estimate was attenuated relative to the primary analysis and the dementia estimate was no longer statistically significant (Model 4: cognitive impairment HR 1.29, 95% CI 1.10 to 1.53; dementia HR 0.85, 95% CI 0.49 to 1.48, *p =* 0.570). Third, in analyses stratified by dental care utilization (Table S12), the edentulism–cognitive impairment association remained significant in both strata (recent visit HR 1.55; no recent visit HR 1.36; interaction *p =* 0.181), whereas the edentulism–dementia association was not significant among participants with a recent dental visit (HR 1.05, *p =* 0.879) but remained significant among those without (HR 1.44, *p =* 0.015; interaction *p <* 0.001). E‐Values for the fully adjusted edentulism–cognition associations were 2.34 for cognitive impairment, 1.88 for dementia, and 2.15 for mortality (Table S13), indicating that moderate unmeasured confounding would be required to fully explain these associations. The edentulism–dementia association was directionally consistent across the three race‐and‐ethnicity‐aware Gianattasio–Power classifications (Hurd algorithm HR 1.38, Expert algorithm HR 1.79, LASSO algorithm HR 1.27, with the LASSO estimate not statistically significant; Table S14), and the multiple‐imputation estimates were consistent with the complete‐case mediation results (Table S15).

## 4 DISCUSSION

In this nationally representative study of 4,238 older adults in the US, we found that edentulism was independently associated with elevated blood‐based markers of neurodegeneration (NfL) and astrocyte reactivity (GFAP), and with increased 10‐year risk of overall cognitive impairment and dementia. Notably, the associations of edentulism with NfL and GFAP persisted after adjustment for a comprehensive set of sociodemographic, behavioral, clinical, and genetic factors, while the associations with pTau‐181 and the Aβ42/40 ratio were null or attenuated after covariate adjustment. Mediation analyses revealed that GFAP partially mediated the edentulism–cognitive impairment association (9.8% mediated) and that both NfL and GFAP mediated the edentulism–dementia association (9.8% and 12.0%, respectively). To our knowledge, this is the first population‐based study to demonstrate that blood‐based neurodegenerative biomarkers partially mediate the pathway from edentulism to cognitive impairment.

Our finding that edentulism was associated with 49% increased risk of cognitive impairment and 28% increased risk of dementia in fully adjusted models is consistent with the broader epidemiological literature.^3^, ^4^, ^5^, ^7^, ^9^, ^34^ The dose–response meta‐analysis reported a pooled HR of 1.48 for cognitive impairment among individuals with tooth loss,^4^ while another reported a pooled HR of 1.23 for dementia.^9^ Studies using HRS data have similarly demonstrated that edentulism predicts steeper cognitive decline over time.^3^, ^5^, ^43^

The selective association of edentulism with NfL and GFAP, but not with pTau‐181 or Aβ42/40, is biologically informative. NfL is a neuron‐specific cytoskeletal protein released after neuroaxonal injury of any etiology, and elevated plasma NfL has been associated with a broad range of neurodegenerative conditions as well as cardiovascular and metabolic comorbidities.^23^, ^44^ GFAP is a marker of astrocyte reactivity, and astrocytes play a central role in neuroinflammatory responses, blood–brain barrier maintenance, and synaptic regulation.^45^ Recent evidence from the BioFINDER‐2 study and other cohorts has demonstrated that plasma GFAP is elevated in preclinical stages of AD and is closely linked to amyloid pathology and subsequent tau accumulation.^24^, ^25^ In contrast, pTau‐181 and Aβ42/40 reflect more specific features of AD neuropathology, including tau hyperphosphorylation and amyloid metabolism, which are less likely to be influenced by peripheral inflammatory exposures.^44^, ^46^ The pattern of our findings is therefore consistent with a model in which edentulism promotes generalized neuroinflammation and neuroaxonal injury, as reflected by elevations in GFAP and NfL, rather than driving AD‐specific amyloid or tau pathology. This interpretation aligns with evidence that chronic peripheral inflammation, such as that arising from periodontal disease, can activate microglia and astrocytes through cytokine‐mediated signaling across the blood‐brain barrier.^12^

The mediation results provide novel quantitative evidence that GFAP and NfL constitute part of the biological pathway through which edentulism influences cognitive outcomes. GFAP mediated 9.8% of the edentulism–cognitive impairment association and 12.0% of the edentulism–dementia association, while NfL mediated 9.8% of the dementia association. Although these proportions are modest, they are noteworthy given the multifactorial nature of dementia and the expectation that any single pathway would account for only a fraction of the total effect.^14^, ^16^ The finding that GFAP, but not NfL, significantly mediated the cognitive impairment outcome may reflect the earlier involvement of astrocyte activation in the neurodegenerative cascade, consistent with evidence that GFAP elevation precedes overt neuroaxonal damage in preclinical disease stages.^47^ The substantial proportion of the association that remained unexplained by these two biomarkers suggests that additional pathways, including nutritional deficiency from impaired masticatory function,^19^ reduced sensory stimulation of the hippocampus via the trigeminal nerve,^10^ physiological dysregulation measured by accelerated biological aging,^14^ and direct microbial invasion of the central nervous system,^11^ likely contribute to the overall edentulism–dementia association.

Dividing by dental care utilization yielded important insights. The edentulism–dementia association was substantially attenuated and was not statistically significant among persons with dental visit within the past 2 years (HR 1.05; Table S12), whereas the edentulism–cognitive impairment association persisted. This finding may reflect the complex role of dental care utilization as both a confounder and a consequence of edentulism. Edentulous individuals are substantially less likely to seek dental care (25.5% versus 70.6% in our sample), and dental care access itself may be a marker of broader health‐seeking behavior, health literacy, and socioeconomic advantage, all of which independently influence dementia risk.^43^, ^48^ The persistence of GFAP‐mediated effects in models that adjusted for dental care utilization (9.8% for cognitive impairment and 12.0% for dementia) suggests that the neuroinflammatory pathway operates at least partly independently of dental care access patterns. The pronounced socioeconomic and racial and ethnic disparities in edentulism observed in our cohort underscore the importance of addressing oral health as a component of health equity strategies aimed at reducing dementia disparities.^6^, ^49^ Taken together, these results caution that upstream socioeconomic and access‐related factors, of which dental care utilization is a marker, may be more central drivers of the observed edentulism–dementia association than tooth loss per se, and that edentulism may partly index cumulative social disadvantage rather than acting solely as an independent causal exposure.^50^

This study has several strengths. We used a large, nationally representative cohort with survey weights to produce population‐representative estimates. The 10‐year follow‐up period enabled examination of long‐term cognitive trajectories. The availability of four blood‐based neurodegenerative biomarkers measured on a validated ultrasensitive platform (Quanterix single molecule array [Simoa]) allowed simultaneous assessment of neuroinflammatory (NfL, GFAP), tau (pTau‐181), and amyloid (Aβ42/40) pathways. The sequential adjustment strategy, competing risk analyses, and multiple sensitivity analyses strengthen the internal validity of the findings.

Several limitations should be acknowledged. First, edentulism was ascertained by self‐report and the timing of tooth loss is unknown; some misclassification is possible, although self‐reported tooth loss has demonstrated acceptable validity in population‐based surveys. Second, the exposure (2012), mediators (2016), and outcomes (2012 to 2022) were measured at different time points; the temporal ordering is consistent with mediation assumptions, but the 4‐year lag between edentulism assessment and biomarker measurement introduces the possibility that intervening factors could have influenced biomarker levels. Relatedly, when the time origin was reset to 2016 in the landmark analysis, the edentulism–dementia association was attenuated to non‐significance after full adjustment, whereas the cognitive impairment association persisted; this likely reflects both the longer and more variable preclinical course of dementia, which the 2016 to 2022 window may be underpowered to capture, and a survival effect, in which participants with co‐occurring edentulism and early dementia were less likely to remain event‐free until the 2016 landmark and were therefore selectively excluded from the landmark sample. Accordingly, the edentulism–dementia association in particular should be interpreted with caution. Third, the biomarkers were measured at a single time point, precluding assessment of longitudinal biomarker trajectories. Fourth, edentulism is a binary measure that does not capture the severity of partial tooth loss or the use of dental prostheses; the dose–response relationship reported elsewhere could not be examined.^4^, ^7^, ^9^ Fifth, although we adjusted for a comprehensive set of confounders, residual confounding by unmeasured factors, such as childhood cognition, lifetime socioeconomic trajectories, or oral hygiene practices, cannot be excluded. Sixth, the Langa‐Weir classification is an algorithmic approach that, while validated against clinical diagnoses,^33^ may misclassify some cases, particularly among racial and ethnic minority groups. Finally, causal mediation analysis with observational data requires strong assumptions, including no unmeasured mediator–outcome confounders, that are inherently untestable.^36^

## 5 CONCLUSIONS

In conclusion, this study provides population‐based evidence that blood‐based markers of astrocyte reactivity (GFAP) and neuroaxonal injury (NfL) account for a proportion of the association between edentulism and cognitive decline in older adults. These observational findings are consistent with a pathway in which oral health deterioration may contribute to neurodegeneration through neuroinflammation, and the attenuation of the dementia association after accounting for dental care utilization suggests that upstream socioeconomic and access‐related factors may be more central than tooth loss itself. The findings support further study of oral health within dementia prevention frameworks, alongside efforts to promote equitable access to dental care. Future longitudinal studies with serial biomarker measurements, clinical dental examinations, and designs better suited to causal inference are needed to determine whether oral health interventions can attenuate neuroinflammatory biomarker elevation and reduce dementia risk.

## AUTHOR CONTRIBUTIONS

X.Q. conceptualized and designed the study, conducted all analyses, and wrote the first draft. R.L. contributed to data preparation and critical revision. D.W.B. and Q.T. provided expertise on biomarker interpretation and critical revision. H.L. contributed to study design and critical revision. Y.L. and Z.X. contributed to data management and critical revision. K.M.L. provided expertise on cognitive outcome classification, the Venous Blood Study biomarker data, and the Health and Retirement Study data. B.W. provided senior oversight, contributed to conceptualization and funding acquisition, and critically revised the manuscript. All authors had written and revised the draft, verified the underlying data, and had final responsibility for the decision to submit for publication.

## CONFLICT OF INTEREST STATEMENT

The authors declare no conflicts of interest. Author disclosures are available in the Supporting Information.

## CONSENT STATEMENT

The HRS protocol was approved by the Institutional Review Board at the University of Michigan (approval number HUM00099822). All participants provided written informed consent for the core interview and the Venous Blood Study. This study was conducted using de‐identified data and was deemed exempt from additional institutional review.

## DECLARATION OF GENERATIVE ARTIFICIAL INTELLIGENCE IN SCIENTIFIC WRITING

We declare that Anthropic's Claude Opus 4.7 was used for language polishing and for assistance in formatting and organizing the statistical analysis code; all code was written, reviewed, executed, and verified by the authors. No artificial intelligence (AI) tool was used to design the study, conduct or interpret the statistical analyses, generate any data or results, or draft the scientific content, conclusions, or interpretation. The authors take full responsibility for the accuracy and integrity of all content, and confirm that this declaration complies with the journal's policies on AI tool usage.

## Supporting information




**Supporting Information**: alz71731‐sup‐0001‐DisclosuresForms.pdf

**Supporting Information**: alz71731‐sup‐0002‐SuppMat.docx

## Data Availability

De‐identified data from the Health and Retirement Study are publicly available through the HRS website (https://hrsdata.isr.umich.edu). The Venous Blood Study biomarker data are available through submitting sensitive health data request to the HRS. The full Stata analysis code supporting this study has been deposited in a public Open Science Framework repository (https://osf.io/9kf7q). The study protocol is available from the corresponding author upon reasonable request.
