# Supplementary material for: Edentulism, blood‐based neurodegenerative biomarkers, and 10‐year dementia risk: A mediation analysis in a nationally representative United States cohort
Source: Alzheimers Dement. 2026 Aug 5;22(8):e71731. doi: 10.1002/alz.71731 (PMC13440353; doi:10.1002/alz.71731)
Supplement: Supplementary file 2 [file ALZ-22-e71731-s001.docx]

**Supplementary Materials**

**Table of contents**

**Table S1. Comparison of included versus excluded participants on 2012 baseline characteristics**

**Figure S1. Directed acyclic graph for the edentulism–cognition relationship**

**Table S2. Association of edentulism with blood-based neurodegenerative biomarkers**

**Table S3. Cox proportional hazards models for edentulism with cognitive outcomes**

**Table S4. Association of edentulism with all-cause mortality**

**Table S5. Cox proportional hazards models with competing risk of death**

**Table S6. Four-way decomposition causal mediation analysis**

**Table S7. Per-analyte distribution, interassay coefficient of variation, and post-hoc power**

**Table S8. APOE ε4 × edentulism interaction on blood-based neurodegenerative biomarkers**

**Table S9. Dementia status × edentulism interaction on blood-based neurodegenerative biomarkers**

**Table S10. Cognitive impairment, not dementia (CIND) × edentulism interaction on blood-based neurodegenerative biomarkers**

**Table S11. Cox proportional hazards models for edentulism with cognitive outcomes, with the time origin reset to the 2016 landmark**

**Table S12. Edentulism–cognitive outcome associations stratified by dental care utilization**

**Table S13. E-value sensitivity analysis for unmeasured confounding**

**Table S14. Edentulism–dementia association under Gianattasio-Power dementia classifications**

**Table S15. Multiple-imputation sensitivity analysis for the mediation models**

**Table S1. Comparison of included versus excluded participants on 2012 baseline characteristics**. P-values from chi-square tests (categorical) or independent t-tests (continuous). The APOE ε4 denominator is restricted to genotyped participants.

| **Characteristic** | **Excluded (N=16,316)** | **Included (N=4,238)** | **Total (N=20,554)** | **P-value** |
| --- | --- | --- | --- | --- |
| **%** | 79.4 | 20.6 | 100 |  |
| **Age, mean (SD)** | 67.27 (12.02) | 65.24 (9.62) | 66.85 (11.60) | <0.001 |
| **Age groups, n (%)** |  |  |  |  |
| ≤64 | 7,581 (46.5%) | 2,228 (52.6%) | 9,809 (47.7%) | <0.001 |
| 65-74 | 3,904 (23.9%) | 1,169 (27.6%) | 5,073 (24.7%) |  |
| 75+ | 4,831 (29.6%) | 841 (19.8%) | 5,672 (27.6%) |  |
| **Female, n (%)** | 9,524 (58.4%) | 2,490 (58.8%) | 12,014 (58.5%) | 0.653 |
| **Race and ethnicity, n (%)** |  |  |  |  |
| Non-Hispanic White | 10,325 (63.3%) | 2,795 (66.0%) | 13,120 (63.8%) | <0.001 |
| Non-Hispanic Black | 3,173 (19.4%) | 712 (16.8%) | 3,885 (18.9%) |  |
| Hispanic | 2,225 (13.6%) | 598 (14.1%) | 2,823 (13.7%) |  |
| Other | 593 (3.6%) | 133 (3.1%) | 726 (3.5%) |  |
| **Married, n (%)** | 10,211 (62.6%) | 2,868 (67.7%) | 13,079 (63.6%) | <0.001 |
| **Education, n (%)** |  |  |  |  |
| Low (≤high school) | 8,780 (53.8%) | 2,113 (49.9%) | 10,893 (53.0%) | <0.001 |
| Mid (some college) | 4,001 (24.5%) | 1,098 (25.9%) | 5,099 (24.8%) |  |
| High (college+) | 3,535 (21.7%) | 1,027 (24.2%) | 4,562 (22.2%) |  |
| **Annual household income, n (%)** |  |  |  |  |
| <$50,000 | 9,868 (60.5%) | 2,322 (54.8%) | 12,190 (59.3%) | <0.001 |
| $50,000–$99,999 | 3,700 (22.7%) | 1,101 (26.0%) | 4,801 (23.4%) |  |
| $100,000–$199,999 | 1,959 (12.0%) | 580 (13.7%) | 2,539 (12.4%) |  |
| ≥$200,000 | 789 (4.8%) | 235 (5.5%) | 1,024 (5.0%) |  |
| **Dental visits past two years, n (%)** | 9,647 (59.3%) | 2,740 (64.7%) | 12,387 (60.4%) | <0.001 |
| **Smoking status, n (%)** |  |  |  |  |
| Never | 7,099 (43.5%) | 1,873 (44.2%) | 8,972 (43.7%) | 0.075 |
| Former | 6,717 (41.2%) | 1,775 (41.9%) | 8,492 (41.3%) |  |
| Current | 2,500 (15.3%) | 590 (13.9%) | 3,090 (15.0%) |  |
| **Exercise (≥1/wk), n (%)** | 10,367 (63.5%) | 3,033 (71.6%) | 13,400 (65.2%) | <0.001 |
| **Obesity (BMI≥30), n (%)** | 5,640 (34.6%) | 1,656 (39.1%) | 7,296 (35.5%) | <0.001 |
| **Depression, n (%)** | 3,591 (22.0%) | 647 (15.3%) | 4,238 (20.6%) | <0.001 |
| **Hypertension, n (%)** | 9,758 (59.8%) | 2,437 (57.5%) | 12,195 (59.3%) | 0.007 |
| **Diabetes, n (%)** | 3,856 (23.6%) | 935 (22.1%) | 4,791 (23.3%) | 0.031 |
| **Heart disease, n (%)** | 3,978 (24.4%) | 849 (20.0%) | 4,827 (23.5%) | <0.001 |
| **Arthritis, n (%)** | 9,165 (56.2%) | 2,376 (56.1%) | 11,541 (56.1%) | 0.910 |
| **Stroke, n (%)** | 1,562 (9.6%) | 292 (6.9%) | 1,854 (9.0%) | <0.001 |
| **APOE ε4 carrier, n (%)** |  |  |  |  |
| No ε4 | 131 (77.1%) | 2,848 (74.4%) | 2,979 (74.5%) | 0.433 |
| Has ε4 | 39 (22.9%) | 981 (25.6%) | 1,020 (25.5%) |  |

**Figure S1. Directed acyclic graph for the edentulism–cognition relationship.** Dental care utilization is modeled as one of the shared common causes (influencing both edentulism and cognitive outcomes via access, health literacy, and socioeconomic position), not as a mediator or collider.

**Table S2. Association of edentulism with blood-based neurodegenerative biomarkers.** β coefficients represent the mean difference in biomarker levels for edentulous versus dentate older adults. Model 1: unadjusted. Model 2: adjusted for age, sex, race and ethnicity, marital status, education, and income. Model 3: Model 2 plus smoking, exercise, dental care utilization, obesity, depression, hypertension, diabetes, heart disease, arthritis, and stroke. Model 4: Model 3 plus APOE ε4 carrier status. All models incorporate Venous Blood Study weights. NfL=neurofilament light chain. GFAP=glial fibrillary acidic protein. pTau-181=phosphorylated tau-181.

| **Biomarker** | **Model** | **β** | **SE** | **95% CI** | **P-value** |
| --- | --- | --- | --- | --- | --- |
| Aβ42/40 Ratio | Model 1 | 0.000 | 0.001 | (-0.002, 0.003) | 0.880 |
|  | Model 2 | 0.000 | 0.001 | (-0.002, 0.003) | 0.755 |
|  | Model 3 | 0.001 | 0.001 | (-0.001, 0.004) | 0.352 |
|  | Model 4 | 0.001 | 0.001 | (-0.001, 0.004) | 0.334 |
| pTau-181 (pg/mL) | Model 1 | 0.40 | 0.13 | (0.14, 0.66) | 0.002 |
|  | Model 2 | 0.19 | 0.13 | (-0.06, 0.45) | 0.140 |
|  | Model 3 | 0.22 | 0.13 | (-0.03, 0.48) | 0.089 |
|  | Model 4 | 0.12 | 0.11 | (-0.09, 0.33) | 0.247 |
| NfL (pg/mL) | Model 1 | 9.39 | 1.67 | (6.12, 12.67) | <0.001 |
|  | Model 2 | 4.81 | 1.55 | (1.77, 7.85) | 0.002 |
|  | Model 3 | 4.68 | 1.50 | (1.73, 7.62) | 0.002 |
|  | Model 4 | 4.21 | 1.59 | (1.08, 7.33) | 0.008 |
| GFAP (pg/mL) | Model 1 | 26.33 | 4.99 | (16.55, 36.11) | <0.001 |
|  | Model 2 | 9.17 | 4.06 | (1.21, 17.12) | 0.024 |
|  | Model 3 | 11.38 | 4.20 | (3.15, 19.60) | 0.007 |
|  | Model 4 | 8.06 | 3.61 | (0.97, 15.15) | 0.026 |

**Table S3. Cox proportional hazards models for edentulism with cognitive outcomes (Health and Retirement Study 2012–2022).** Incident overall cognitive impairment: among participants with normal cognition at baseline (n=3,519). Incident dementia: among participants with normal cognition or CIND at baseline (n=4,150). Model 1: unadjusted. Model 2: adjusted for age, sex, race and ethnicity, marital status, education, and income. Model 3: Model 2 plus smoking, exercise, dental care utilization, obesity, depression, hypertension, diabetes, heart disease, arthritis, and stroke. Model 4: Model 3 plus APOE ε4 carrier status. All models incorporate Venous Blood Study weights.

| **Models** | **N** | **Events** | **Hazard Ratio** | **95% Confidence Intervals** | **P-value** |
| --- | --- | --- | --- | --- | --- |
| **Incident cognitive impairment** |  |  |  |  |  |
| Model 1 | 3,519 | 1,163 | 2.03 | (1.77, 2.33) | <0.001 |
| Model 2 | 3,519 | 1,163 | 1.57 | (1.29, 1.91) | <0.001 |
| Model 3 | 3,519 | 1,163 | 1.52 | (1.24, 1.86) | <0.001 |
| Model 4 | 3,189 | 1,052 | 1.49 | (1.20, 1.85) | <0.001 |
| **Incident dementia** |  |  |  |  |  |
| Model 1 | 4,150 | 455 | 2.04 | (1.62, 2.57) | <0.001 |
| Model 2 | 4,150 | 455 | 1.46 | (1.07, 1.98) | 0.017 |
| Model 3 | 4,150 | 455 | 1.42 | (1.04, 1.94) | 0.026 |
| Model 4 | 3,749 | 414 | 1.28 | (1.03, 1.59) | 0.025 |

**Table S4. Association of edentulism with all-cause mortality.** Normal cognition or CIND at baseline (n=4,150). Model 1: unadjusted. Model 2: adjusted for age, sex, race and ethnicity, marital status, education, and income. Model 3: Model 2 plus smoking, exercise, dental care utilization, obesity, depression, hypertension, diabetes, heart disease, arthritis, and stroke. Model 4: Model 3 plus APOE ε4 carrier status. All models incorporate Venous Blood Study weights.

| **Model** | **N** | **Events** | **Hazard Ratio** | **95% Confidence Intervals** | **P-value** |
| --- | --- | --- | --- | --- | --- |
| Model 1 | 4,150 | 732 | 2.56 | (2.07, 3.17) | <0.001 |
| Model 2 | 4,150 | 732 | 1.62 | (1.35, 1.94) | <0.001 |
| Model 3 | 4,150 | 732 | 1.46 | (1.16, 1.84) | <0.001 |
| Model 4 | 3,749 | 681 | 1.40 | (1.12, 1.75) | <0.001 |

**Table S5. Cox proportional hazards models with competing risk of death (cause-specific hazard), Health and Retirement Study 2012–2022.** Normal cognition at baseline (outcome: incident overall cognitive impairment) or normal cognition/CIND at baseline (outcome: incident dementia). Competing deaths=participants who died without experiencing the cognitive outcome. Model 1: unadjusted. Model 2: adjusted for age, sex, race and ethnicity, marital status, education, and income. Model 3: Model 2 plus smoking, exercise, dental care utilization, obesity, depression, hypertension, diabetes, heart disease, arthritis, and stroke. Model 4: Model 3 plus APOE ε4 carrier status. All models incorporate HRS Venous Blood Study weights.

| **Model** | **Cause-Specific**  **HR (95% CI)** | **P-value** | **Fine-Gray Competing Risk**  **HR (95% CI)** | **P-value** |
| --- | --- | --- | --- | --- |
| **Incident cognitive impairment** |  |  |  |  |
| Model 1 | 2.03 (1.67, 2.47) | <0.001 | 1.96 (1.62, 2.39) | <0.001 |
| Model 2 | 1.32 (1.09, 1.60) | 0.005 | 1.29 (1.06, 1.57) | 0.012 |
| Model 3 | 1.24 (1.04, 1.48) | 0.017 | 1.22 (1.09, 1.37) | <0.001 |
| Model 4 | 1.22 (1.02, 1.46) | 0.030 | 1.19 (1.06, 1.34) | 0.004 |
| **Incident dementia** |  |  |  |  |
| Model 1 | 2.04 (1.52, 2.74) | <0.001 | 1.89 (1.41, 2.54) | <0.001 |
| Model 2 | 1.46 (1.11, 1.91) | 0.007 | 1.36 (1.08, 1.71) | 0.008 |
| Model 3 | 1.42 (1.07, 1.89) | 0.016 | 1.34 (1.09, 1.65) | 0.005 |
| Model 4 | 1.28 (1.08, 1.51) | 0.004 | 1.22 (1.03, 1.45) | 0.021 |

**Table S6. Four-way decomposition causal mediation analysis (med4way) of NfL and GFAP as mediators of edentulism–cognitive outcome associations.** The total effect is decomposed into the controlled direct effect (CDE), the reference interaction (INTref), the mediated interaction (INTmed), and the pure indirect effect (PIE); the natural direct effect (NDE) equals CDE plus INTref and the natural indirect effect (NIE) equals INTmed plus PIE. Effects are on the excess hazard-ratio scale; mediators are standardized per 1-SD. Bootstrap 95% confidence intervals (1,000 replications). All models adjust for age, sex, race and ethnicity, marital status, education, income, smoking, exercise, dental care utilization, obesity, depression, hypertension, diabetes, heart disease, arthritis, stroke, and APOE ε4, with Venous Blood Study weights.

| **Outcome / Mediator** | **CDE** | **INTref** | **INTmed** | **PIE** | **Total HR (95% CI)** | **% mediated** | **Path a beta (p)** | **Path b HR (p)** |
| --- | --- | --- | --- | --- | --- | --- | --- | --- |
| **Incident cognitive impairment** |  |  |  |  |  |  |  |  |
| NfL | 0.49 | 0.04 | -0.01 | 0.00 | 1.52 (1.24, 1.86) | -1.6 | 1.521 (0.269) | 1.0042 (<0.001) |
| GFAP | 0.40 | 0.07 | 0.04 | 0.01 | 1.52 (1.24, 1.86) | 9.8 | 8.234 (0.031) | 1.0022 (<0.001) |
| **Incident dementia** |  |  |  |  |  |  |  |  |
| NfL | 0.32 | 0.06 | 0.04 | 0.00 | 1.42 (1.04, 1.94) | 9.8 | 3.998 (0.005) | 1.0039 (<0.001) |
| GFAP | 0.30 | 0.07 | 0.05 | 0.00 | 1.42 (1.04, 1.94) | 12.0 | 10.118 (0.016) | 1.0025 (<0.001) |

**Table S7. Per-analyte distribution, interassay coefficient of variation, and post-hoc power.** All four biomarkers were right-skewed (raw skewness 2.4 to 11.6) and were natural-log transformed for regression; log transformation substantially reduced skewness (to within ±0.6 for all analytes). Interassay coefficients of variation are reported per analyte from the Health and Retirement Study Venous Blood Study quality-control documentation (overall range 8.55% to 13.34%). Post-hoc power reflects the minimum detectable standardized mean difference (Cohen’s d) between edentulous and dentate participants at α=0.05 and 80% power, given the observed assay subsample sizes.

| **Analyte** | **Median (IQR)** | **Skewness (raw/log)** | **Interassay CV** | **Min. detectable d** | **N** |
| --- | --- | --- | --- | --- | --- |
| Aβ42/40 ratio | 0.065 (0.056, 0.073) | 8.8 / 0.30 | 9.4% | 0.13 | 4,166 |
| pTau-181 (pg/mL) | 1.57 (1.06, 2.43) | 7.7 / −0.14 | 11.2% | 0.13 | 4,138 |
| NfL (pg/mL) | 17.7 (11.7, 28.9) | 11.6 / 0.61 | 8.6% | – | 4,167 |
| GFAP (pg/mL) | 85.9 (55.8, 132.2) | 2.4 / 0.04 | 13.3% | – | 4,167 |

**Table S8. APOE ε4 × edentulism interaction on blood-based neurodegenerative biomarkers.** Interaction terms test whether the association of edentulism with biomarkers differs by APOE ε4 status. All models adjusted for Model 3 covariates: age, sex, race and ethnicity, marital status, education, income, smoking, exercise, dental care utilization, obesity, depression, hypertension, diabetes, heart disease, arthritis, and stroke. All models incorporate Venous Blood Study weights. NfL=neurofilament light chain. GFAP=glial fibrillary acidic protein. pTau-181=phosphorylated tau-181.

| **Biomarker** | **Term** | **β (95% CI)** | **P-value** |
| --- | --- | --- | --- |
| Aβ42/40 Ratio | Edentulism | 0.001 (-0.002, 0.005) | 0.389 |
|  | APOE ε4 (vs non-carrier) | -0.004 (-0.007, -0.002) | 0.001 |
|  | Edentulism × APOE ε4 | -0.000 (-0.006, 0.005) | 0.871 |
| pTau-181 (pg/mL) | Edentulism | 0.11 (-0.14, 0.35) | 0.395 |
|  | APOE ε4 (vs non-carrier) | 0.25 (0.08, 0.43) | 0.005 |
|  | Edentulism × APOE ε4 | 0.07 (-0.40, 0.51) | 0.763 |
| NfL (pg/mL) | Edentulism | 4.94 (1.10, 8.77) | 0.012 |
|  | APOE ε4 (vs non-carrier) | 0.51 (-1.54, 2.55) | 0.628 |
|  | Edentulism × APOE ε4 | -2.77 (-8.56, 3.01) | 0.348 |
| GFAP (pg/mL) | Edentulism | 10.42 (2.33, 18.50) | 0.012 |
|  | APOE ε4 (vs non-carrier) | 14.40 (8.22, 20.58) | <0.001 |
|  | Edentulism × APOE ε4 | -8.98 (-23.80, 5.83) | 0.235 |

**Table S9. Dementia status × edentulism interaction on blood-based neurodegenerative biomarkers.** Dementia=classified as demented at any wave from 2012 to 2016. Interaction terms test whether the edentulism–biomarker association differs by dementia status. All models adjusted for Model 3 covariates: age, sex, race and ethnicity, marital status, education, income, smoking, exercise, dental care utilization, obesity, depression, hypertension, diabetes, heart disease, arthritis, and stroke. All models incorporate Venous Blood Study weights. NfL=neurofilament light chain. GFAP=glial fibrillary acidic protein. pTau-181=phosphorylated tau-181.

| **Biomarker** | **Term** | **β (95% CI)** | **P-value** |
| --- | --- | --- | --- |
| Aβ42/40 Ratio | Edentulism | 0.002 (-0.001, 0.005) | 0.183 |
|  | Dementia (vs non-dementia) | 0.005 (0.001, 0.010) | 0.028 |
|  | Edentulism × Dementia | -0.007 (-0.014, -0.001) | 0.038 |
| pTau-181 (pg/mL) | Edentulism | 0.17 (-0.09, 0.43) | 0.194 |
|  | Dementia (vs non-dementia) | 0.16 (-0.14, 0.45) | 0.295 |
|  | Edentulism × Dementia | 0.47 (-0.55, 1.49) | 0.367 |
| NfL (pg/mL) | Edentulism | 3.51 (0.71, 6.30) | 0.014 |
|  | Dementia (vs non-dementia) | 2.98 (-1.53, 7.48) | 0.195 |
|  | Edentulism × Dementia | 10.83 (-3.99, 25.66) | 0.152 |
| GFAP (pg/mL) | Edentulism | 8.05 (1.32, 14.78) | 0.019 |
|  | Dementia (vs non-dementia) | 17.66 (3.68, 31.62) | 0.013 |
|  | Edentulism × Dementia | 28.99 (-18.13, 76.11) | 0.228 |

**Table S10. Cognitive impairment, not dementia (CIND) × edentulism interaction on blood-based neurodegenerative biomarkers.** CIND=classified as CIND at any wave from 2012 to 2016. Interaction terms test whether the edentulism–biomarker association differs by CIND status. All models adjusted for Model 3 covariates: age, sex, race and ethnicity, marital status, education, income, smoking, exercise, dental care utilization, obesity, depression, hypertension, diabetes, heart disease, arthritis, and stroke. All models incorporate Venous Blood Study weights. NfL=neurofilament light chain. GFAP=glial fibrillary acidic protein. pTau-181=phosphorylated tau-181.

| **Biomarker** | **Term** | **β (95% CI)** | **P-value** |
| --- | --- | --- | --- |
| Aβ42/40 Ratio | Edentulism | 0.001 (-0.002, 0.004) | 0.387 |
|  | CIND (vs normal) | 0.001 (-0.001, 0.003) | 0.585 |
|  | Edentulism × CIND | 0.002 (-0.004, 0.008) | 0.546 |
| pTau-181 (pg/mL) | Edentulism | 0.15 (-0.20, 0.49) | 0.405 |
|  | CIND (vs normal) | 0.18 (0.01, 0.36) | 0.046 |
|  | Edentulism × CIND | -0.02 (-0.48, 0.51) | 0.951 |
| NfL (pg/mL) | Edentulism | 2.25 (-0.61, 5.11) | 0.123 |
|  | CIND (vs normal) | 4.13 (1.34, 6.92) | 0.004 |
|  | Edentulism × CIND | 2.31 (-4.44, 9.06) | 0.502 |
| GFAP (pg/mL) | Edentulism | 9.74 (1.59, 17.89) | 0.019 |
|  | CIND (vs normal) | 13.32 (4.74, 21.89) | 0.002 |
|  | Edentulism × CIND | -8.39 (-22.87, 6.10) | 0.256 |

**Table S11. Cox proportional hazards models for edentulism with cognitive outcomes, with the time origin reset to the 2016 landmark (the biomarker measurement wave).** Model 1: unadjusted. Model 2: adjusted for age, sex, race and ethnicity, marital status, education, and income. Model 3: Model 2 plus smoking, exercise, dental care utilization, obesity, depression, hypertension, diabetes, heart disease, arthritis, and stroke. Model 4: Model 3 plus APOE ε4 carrier status. All models incorporate HRS Venous Blood Study weights.

| **Model** | **N** | **Events** | **Hazard Ratio** | **95% Confidence Intervals** | **P-value** |
| --- | --- | --- | --- | --- | --- |
| **Incident cognitive impairment** |  |  |  |  |  |
| Model 1 | 3,100 | 651 | 2.03 | (1.67, 2.47) | <0.001 |
| Model 2 | 3,100 | 651 | 1.66 | (1.29, 2.14) | <0.001 |
| Model 3 | 3,100 | 651 | 1.32 | (1.14, 1.53) | <0.001 |
| Model 4 | 2,824 | 612 | 1.29 | (1.10, 1.53) | 0.002 |
| **Incident dementia** |  |  |  |  |  |
| Model 1 | 4,031 | 308 | 2.04 | (1.52, 2.74) | <0.001 |
| Model 2 | 4,031 | 308 | 1.02 | (0.64, 1.63) | 0.922 |
| Model 3 | 4,031 | 308 | 0.90 | (0.55, 1.49) | 0.691 |
| Model 4 | 3,678 | 286 | 0.85 | (0.49, 1.48) | 0.570 |

**Table S12. Edentulism–cognitive outcome associations stratified by dental care utilization.** Models adjust for age, sex, race and ethnicity, marital status, education, income, smoking, exercise, obesity, depression, hypertension, diabetes, heart disease, arthritis, stroke, and APOE ε4 (dental care not included as a covariate within strata). All models incorporate HRS Venous Blood Study weights.

| **Outcome** | **Recent dental visit** | | **No recent visit** | | **Interaction P-value** |
| --- | --- | --- | --- | --- | --- |
|  | **HR (95% CI)** | **P-value** | **HR (95% CI)** | **P-value** |  |
| Incident cognitive impairment | 1.55 (1.14, 1.85) | <0.001 | 1.36 (1.10, 1.68) | 0.004 | 0.181 |
| Incident dementia | 1.05 (0.59, 1.87) | 0.879 | 1.44 (1.07, 1.93) | 0.015 | <0.001 |

**Table S13. E-value sensitivity analysis for unmeasured confounding.** E-values quantify the minimum strength of association, on the risk-ratio scale, that an unmeasured confounder would need with both the exposure (or mediator) and the outcome, conditional on measured covariates, to fully explain the observed association. The E-value for the confidence-interval limit closest to the null is also reported. Total edentulism-outcome E-values are computed from the fully adjusted (Model 4) hazard ratios. Mediator-outcome (path b) E-values are computed on the per-1-SD scale.

| **Association** | **HR (95% CI)** | **E-value (point)** | **E-value (CI limit)** |
| --- | --- | --- | --- |
| **Total exposure-outcome effect** |  |  |  |
| Edentulism → incident cognitive impairment | 1.49 (1.20, 1.85) | 2.34 | 1.69 |
| Edentulism → incident dementia | 1.28 (1.03, 1.59) | 1.88 | 1.21 |
| Edentulism → all-cause mortality | 1.40 (1.12, 1.75) | 2.15 | 1.43 |
| **Mediator-outcome effect, per 1-SD (path b)** |  |  |  |
| NfL → incident cognitive impairment | 1.16 (1.10, 1.22) | 1.59 | 1.44 |
| GFAP → incident cognitive impairment | 1.19 (1.07, 1.33) | 1.67 | 1.34 |
| NfL → incident dementia | 1.14 (1.07, 1.22) | 1.55 | 1.36 |
| GFAP → incident dementia | 1.20 (1.04, 1.38) | 1.69 | 1.24 |

**Table S14. Edentulism–dementia association under race-and-ethnicity-aware Gianattasio-Power dementia classifications.** Three algorithms are reported: Hurd, Expert, and LASSO on HRS participants aged 70+ from 2012 to 2020. Incident dementia is defined among participants not classified as demented at baseline (2012) under the respective algorithm. Model 1: unadjusted. Model 2: adjusted for age, sex, race and ethnicity, marital status, education, and income. Model 3: Model 2 plus smoking, exercise, dental care utilization, obesity, depression, hypertension, diabetes, heart disease, arthritis, and stroke. Model 4: Model 3 plus APOE ε4 carrier status.

| **Algorithm** | **N** | **Events** | **HR (95% CI)** | **P-value** |
| --- | --- | --- | --- | --- |
| Hurd | 1,349 | 336 | 1.38 (1.18, 1.62) | <0.001 |
| Expert | 1,329 | 387 | 1.79 (1.11, 2.89) | 0.018 |
| LASSO | 1,297 | 323 | 1.27 (0.80, 2.02) | 0.316 |

**Table S15. Multiple-imputation sensitivity analysis for the mediation models.** Within the analytic sample, item-level covariate missingness was limited to APOE ε4 carrier status (9.7%) and obesity derived from body mass index (1.2%); all other covariates were complete. Twenty imputations were generated by chained equations (logistic models for the binary covariates), and the Model 4 mediation estimates were pooled using Rubin’s rules. The complete-case estimates are shown alongside the imputed estimates for comparison.

| **Outcome / Mediator** | **Analysis** | **Total effect** | | **Direct effect** | | **% Mediated** |
| --- | --- | --- | --- | --- | --- | --- |
|  |  | **HR (95% CI)** | **P-value** | **HR (95% CI)** | **P-value** |  |
| **Incident cognitive impairment** |  |  |  |  |  |  |
| GFAP | Complete case | 1.52 (1.24, 1.86) | <0.001 | 1.47 (1.20, 1.80) | <0.001 | 9.8 |
| GFAP | Multiple imputation | 1.60 (1.34, 1.91) | <0.001 | 1.54 (1.29, 1.83) | <0.001 | 8.6 |
| **Incident dementia** |  |  |  |  |  |  |
| NfL | Complete case | 1.42 (1.04, 1.94) | 0.027 | 1.38 (1.04, 1.86) | 0.030 | 9.8 |
| NfL | Multiple imputation | 1.55 (1.16, 2.07) | 0.003 | 1.49 (1.11, 1.99) | 0.007 | 9.1 |
| GFAP | Complete case | 1.42 (1.04, 1.94) | 0.027 | 1.37 (1.03, 1.82) | 0.030 | 12.0 |
| GFAP | Multiple imputation | 1.45 (1.06, 1.98) | 0.020 | 1.40 (1.02, 1.91) | 0.036 | 10.2 |
